# Extending the ophthalmological phenotype of Galloway-Mowat syndrome with distinct retinal dysfunction: a report and review of ocular findings

**DOI:** 10.1186/s12886-018-0820-4

**Published:** 2018-06-22

**Authors:** Maha A. Al-Rakan, Manal D. Abothnain, Muhammad T. Alrifai, Majid Alfadhel

**Affiliations:** 1 Deapartment of Clinical Laboratory Sciences, College of Applied Medical Sciences, King Saud University, Riyadh, Saudi Arabia; 2 Division of Neurology, Department of Pediatrics, King Saud bin Abdulaziz University for Health Sciences, King Abdullah International Medical Research Centre, King Abdullah Specialized Children’s Hospital, King Abdulaziz Medical City, Ministry of National Guard-Health Affairs (NGHA), Riyadh, Saudi Arabia; 3 Division of Genetics, Department of Pediatrics, King Saud bin Abdulaziz University for Health Sciences, King Abdullah International Medical Research Centre, King Abdullah Specialized Children Hospital, King Abdulaziz Medical City, Ministry of National Guard-Health Affairs (NGHA), PO Box 22490, Riyadh, 11426 Saudi Arabia

**Keywords:** Galloway-Mowat syndrome, WDR37, Retinal dysfunction, Absence of perinatal and neonatal renal dysfunction

## Abstract

**Background:**

Galloway-Mowat syndrome (GMS) is a rare autosomal recessive condition first described in 1968 and characterized by microcephaly and infantile onset of central nervous system (CNS) abnormalities resulting in severely delayed psychomotor development, cerebellar atrophy, epilepsy, and ataxia, as well as renal abnormalities such as nephrotic syndrome, proteinuria, end-stage renal disease (ESRD), and hiatal hernia.

**Case presentation:**

We describe a GMS case diagnosed with homozygous missense mutation in the *WDR73* gene, with absence of renal abnormalities. We expanded the clinical phenotype of GMS with *WDR73* gene defect to include retinal dysfunction with missense mutation and developmental dysplasia of the hip. We compared eye findings of our case to previously reported cases, and we present an electroretinogram (ERG) picture for the first time in the literature.

**Conclusion:**

We recommend that clinicians screen patients with GM syndrome for retinal dysfunction and that a skeletal survey should be done to detect developmental dysplasia of the hip (DDH) so as to provide for early intervention.

## Background

Galloway-Mowat syndrome (GMS; OMIM 251300) is a rare autosomal recessive genetic neurodegenerative disorder characterized by a classical triad of central nervous system (CNS) abnormalities, including infantile onset of microcephaly, cerebellar atrophy, epilepsy, ataxia, delayed psychomotor development, renal abnormalities (nephrotic syndrome, proteinuria, and end stage renal disease or ESRD), and hiatal hernia. However, hiatal hernia is not an essential sign for diagnosis [1].

Since 1968, approximately 80 GMS cases have been reported, resulting in significant expansion of the heterogeneity of the clinical features to now exclude hiatal hernia [2]. Subsequently, GMS cases were recognized in the absence of renal involvement [3]. As a result of this clinical variability, the syndrome was thought to be genetically heterogeneous. A number of genes were implicated until the *WDR73* gene was mapped to chromosome 15q24-q26 in a family that presented with GMS clinical features [4, 5]. Thereafter, *WDR73* mutations were identified as the causative mutations for GMS using Sanger sequencing and whole exome sequencing (WES) [1, 6–8]. Furthermore, the role of *WDR73* in neurodevelopment has been shown by gene expression and loss of function studies carried out in the zebrafish model system [8]. The *WRD73* gene encodes a multiple WD40 repeats protein with motifs that contain 40–60 amino acids. WD40 proteins function as protein-protein interaction adaptors; these interchangeable substrate receptors target different substrates selectively, provide for signal transduction, transcription regulation for cell cycle control, autophagy, and apoptosis in several different cellular processes [9]. WDR73 protein is expressed in brain and kidney tissue and plays a crucial role in the maintenance of cell architecture and survival [1, 6]. In comparison to GMS cases with negative *WDR73*-mutations, GMS patients with *WDR73*-mutations present with classical GMS clinical features including cerebellar atrophy, thin corpus callosum, brain-stem hypoplasia, occasional coarse face, late-onset and mostly slow progressive nephrotic syndrome. Frequent epileptic symptoms were limited to *WDR73*-positive mutation patients but hiatal hernia was absent in this group [8]. The ophthalmological findings associated with GMS include optic atrophy, nystagmus, strabismus, oculomotor apraxia, myopia, microphthalmia, and corneal opacities [1, 6–8]. Retinal involvement has not been described previously [1].

In the present report, we describe a 41-month-old Saudi female who was diagnosed with GMS that was confirmed by finding a known homozygous missense mutation in the *WDR73* gene. Additionally, we expanded the clinical features of GMS with *WDR73*-mutation to include retinal dysfunction with the missense mutation. We compared eye findings of our case to previously reported cases and present an electroretinogram (ERG) for the first time in the literature.

## Case presentation

A Saudi female infant was delivered by C-section after pre-term labor at 32 weeks. She was a product of in vitro fertilization (IVF) from first-cousin healthy parents, and had a birth weight of 1085 g (<3rd percentile), a length of 46 cm (85th percentile), and a head circumference of 31.5 cm (75th percentile), She had a healthy twin brother and a healthy elder sister. Neonatal examination showed that the patient was active, moving all 4 limbs, and that her primitive reflexes were positive. She was admitted to the intermediate care nursery (ICN) for 17 days for respiratory distress syndrome (RDS) and prematurity.

At the age of 18 months, the patient was presented to our genetics clinic for global developmental delay corresponding to an age of 3 months, hypotonia, upward posture of the big toe, and overlapping 1st and 2nd toes bilaterally. In addition, she had poor vision and evidence of delayed myelination on neuroimaging.

**Fig. 1:**
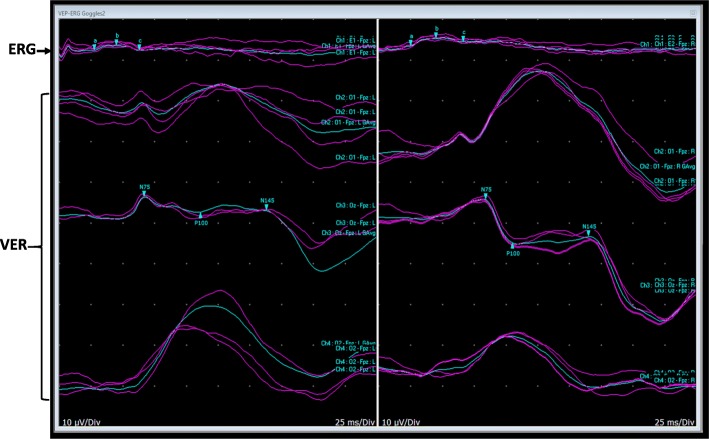
Combined assessment of the patient eyes obtained via electroretinogram (ERG) and visual evoked responses (VER) tests: The upper tracing is a flash ERG (25 ms) stimulus that shows a poorly-formed low amplitude b-wave, and the other tracings below are flash VER with reproducible positive waves at about 100 ms (P100) upon stimulation of either eye. These findings are suggestive of significant retinal dysfunction

At 22 months of age, she could not visually track or fix, sit, grasp, or creep, and she demanded prolonged feeding but did not choke. She had strabismus, horizontal nystagmus, and persistent axial hypotonia. She had abdominal distention with no organomegaly. The right hip demonstrated features of developmental dysplasia of the hip (DDH) with a shallow acetabular roof, a smaller right femoral epiphysis than on the left side, and a dislocated hip with frog like posture of the legs. She responded to sound and appreciated light to her eyes. She had mild to moderate disc pallor and optic atrophy, with visual evoked responses (VER) and ERGs suggesting significant retinal dysfunction (Fig. 1). Magnetic resonance imaging (MRI) revealed high signal intensities of the white matter bilaterally that related to delayed myelination.

Developmentally, at the age of 41 months she had a momentary head control. However, she could not grasp, her fist was open at all times, and she could not fix or follow or sit without support. She had no speech but socially she was able to laugh and smile. Developmentally, she was functioning at the level of a 3-month-old child. On examination, we observed dysmorphic features (i.e., hypertelorism, ptosis, epicanthal folds, micrognathia, long eye lashes and hirsutism, microcephaly, generalized hypotonia, global developmental delays, and impaired vision. Of note, her BUN levels ranged between 0.7 and 3.5 mmol/l (2.5–6) while those of creatinine ranged between 30 and 57 umol/l (27–62), and albumin was 30 g/l >(28–44). All were within normal ranges, with no proteinuria. There were normal urine magnesium levels and protein/creatinine ratios. Extensive laboratory investigations showed normal female karyotype, acylcarnitine profile, plasma amino acids, urinary organic acids, carbohydrate-deficient transferrin (CDT) levels, and very long chain fatty acids (VLCFA), oligosaccharides and mucopolysaccharides. The comparative genomic hybridization (CGH) microarray and DNA molecular testing for *SMN1* gene were unremarkable. Therefore, aminoacidopathies, organic acidemia, fatty acid oxidation defects, congenital disorder of glycosylation, peroxisomal disorders, oligosaccharidosis, and mucopolysaccharidosis were unlikely.

Whole-exome sequencing (WES) revealed that she had a previously described homozygous mutation in the *WDR73* gene [NM_032856.3, c.287G > A (p. Arg96Lys)] which segregated well with the family as the parents and her sister were heterozygous for the same mutation [1].

## Discussion

Table 1 summarizes the ophthalmological findings of GMS. The most common eye abnormalities are optic atrophy (70%), followed by nystagmus (49%) and strabismus (16%), respectively. Hypertelorism and epicanthal folds are present in 8% of the cases, and ptosis, oculomotor apraxia, and microphthalmia occur in approximately 4%. Other rare anomalies include retinopathy, hypoplastic changes of the iris, and corneal opacification.

**Table 1:** Ophthalmological findings of patients with Galloway-Mowat Syndrome [1–8, 10–13, 15–17]

Clinical feature	Number of affected (total: 76)	%
Optic atrophy	53	70%
Nystagmus	37	49%
Strabismus	12	16%
Hypertelorism	7	8%
Epicanthal folds	7	8%
Ptosis	3	4%
Oculomotor apraxia	3	4%
Microphthalmia	3	4%
Retinopathy	3	4%
Hypoplasia of the iris	1	1.3%
Corneal opacification	1	1.3%

In their original report of this syndrome in 1968, Galloway and Mowat described two siblings (one boy, one girl) who suffered from microcephaly, hiatal hernia, and nephrotic syndrome [10]. However, there were no ophthalmologic findings in this family. Subsequently, a series of cases were reported with eye findings [1, 2, 4–8].

Of note, retinopathy was reported previously in association with the c.400_401delAG, p.(Trp136Alafs*2) mutation in the *WDR73* gene [1]. However, we are unaware of any other reports of retinal dysfunction in GMS with a c.287G > A (p. Arg96Lys) WDR*73*-mutation and we delineated the retinal dysfunction phenotype further with ERG (Fig. 1), which showed poorly formed low-amplitude b-waves suggestive of retinal dysfunction. Diagnosis of retinal abnormalities could have been missed previously as several patients died within the first years of life and their visual impairment might have been attributed to optic atrophy, which is a common finding [1].

The mechanisms for the ocular features of this syndrome have not been fully defined. *WDR73* gene expression was highest in the cerebellar cortex, with lesser expression in other brain regions including the neocortex. Additionally, it was expressed in renal glomeruli [1, 6]. *WDR73* mutations may cause disruption in mitosis by interfering with spindle functions [7, 11], which can lead to brain anomalies including ocular lesions. However, such conclusions are hypothetical and functional studies should be done in order to delineate the mechanisms of the ocular findings and other clinical features of this syndrome.

Interestingly, the patient did not have perinatal renal dysfunction or a hiatal hernia, corroborating previous reports [1, 3, 8, 12]. Nonetheless, these features could develop later. In addition, the patient suffered from DDH with a shallow acetabular roof. This finding is novel and not previously described but could be explained by hypotonia. The reported skeletal features of GMS include joint contractures, talipes equinovarus (clubfoot), and camptodactyly [6, 13]. The present case did not manifest these features.

Brain and CNS anomalies are predominant in GMS, and include psychomotor retardation in the form of lack of speech and non-communication, not fixing or following, delayed milestones (90%); hypotonia (80%); microcephaly (80%); and epilepsy (40%) [1, 6, 11]. Other features include ataxia and dystonia [4, 5]. Of note, our patient was severely developmentally delayed and at 3.5 years of age functioned at the level of a 3-month-old infant. Additionally, she had poor white matter myelination on her brain MRI studies. Other possible radiological findings included brain atrophy, with cerebellar and basal ganglia involvement, gyration defects, hypomyelination, a thin corpus callosum, and Dandy-Walker malformation [1, 6–8, 10].

GMS is clinically and genetically heterogeneous. The age of diagnosis has varied from 19 days to 31 years. Most affected individuals died in their teenage years, with common causes of death being nephrotic syndrome and renal failure [7, 11]. Our patient is alive at 41 months, and does not have renal manifestations yet.

There is no genotype-phenotype correlation. All types of mutations have been reported including missense, non-sense, and frameshift deletions [1, 8]. Braun et al. [14] reported a complete list of mutations in 4 genes in *OSGEP, TP53RK, TPRKB,* or *LAGE3*, encoding the 4 subunits of the KEOPS complex in 33 probands of 30 families with GMS with nephrotic syndrome and primary microcephaly. The findings of microcephaly, reduced gyration, and diffuse cortical atrophy may explain the ocular findings in these patients [14].

## Conclusions

In summary, we delineated retinal dysfunction associated with missense mutation supported by ERG findings. Additionally, we described DDH with a shallow acetabular roof as a novel finding in GMS. We suggest that clinicians should screen any patient with GMS for retinal dysfunction, and that a skeletal survey should be done to evaluate for presence of DDH, which if present can benefit from early intervention. Finally, the treatment for this syndrome is supportive and counseling of the parents has to be performed very carefully to avoid raising false hopes.

## Abbreviations

CDT: Carbohydrate deficient transferrin
CNS: Central nervous system
DDH: Developmental dysplasia of the hip
ERG: Electroretinogram
ESRD: End stage renal disease
GMS: Galloway-Mowat syndrome
ICN: Intermediate care nursery
IVF: In vitro fertilization
MRI: Magnetic resonance imaging
RDS: Respiratory distress syndrome
SMA: Spinal muscular atrophy
TMS: Tandem mass spectrometry
VER: Visual evoked response
VLCFA: Very long chain fatty acids
WES: Whole exome sequencing
